# Central Pancreatectomy as a Good Solution in Frantz Tumor Resection: A Case Report

**DOI:** 10.1097/MD.0000000000001165

**Published:** 2015-07-24

**Authors:** Pawel Nachulewicz, Blażej Rogowski, Marcin Obel, Janusz Woźniak

**Affiliations:** From the Clinic of Paediatric Surgery and Traumatology, Medical University of Lublin, Lublin, Poland.

## Abstract

Solid pseudopapillary tumors of the pancreas located in the head or body are a challenging clinical problem because they usually demand extensive surgical procedures, and in most reported cases pancreaticoduodenectomy is the operation of choice in such a location. An alternative method of surgery in precisely selected patients is a procedure known as central pancreatectomy.

The authors present the case of a 13-year-old girl with a 5 cm tumor located in the body of the pancreas. The favorable anatomical location of the tumor suggested central pancreatic resection. The tumor was excised with 1 cm oncologic margins from both sides, and the distal remnant of the pancreas was protected with a Roux-en-Y loop. In the postoperative period the patient required reoperation because of intensive bleeding in the resection site but the duodenal loop was saved and the patient protected from biliary tract reconstruction and exocrine and endocrine insufficiency.

Progress in pancreatic surgery, especially in children, allows less radical options for the reason that preservation of endocrine and exocrine function is very important and protects them, especially from insulin-dependent diabetes in the future.

## INTRODUCTION

Solid pseudopapillary neoplasm of the pancreas (synonyms: solid cystic or papillary cystic tumor, Frantz tumor, solid and papillary epithelial neoplasm) is an uncommon but distinct pancreatic neoplasm with low-metastatic potential.

It accounts for 1% to 3% of all pancreatic malignancies, and the overall mortality rate of the tumor has been estimated to be around 2%. Usually, 90% of patients are females, and 85% of them are under 30-years old, with a median age of 26 years. About 10% of cases occur in men.^1,2^

Solid pseudopapillary tumors of the pancreas were first described by Frantz in 1959. To date, something over 600 cases has been described in the literature.

The tumor may occur anywhere in the pancreas and presents macroscopically as a round, well-demarcated lesion measuring 2 to 17 cm in diameter (average 8 cm).

The tumor is estimated as a low-grade malignant papillary-cystic neoplasm of the pancreas of unclear histogenetic origin, but with highly characteristic, distinct histology, and usually quite benign biological behavior.^2^

Clinical presentation is vague and noncharacteristic. This neoplasm is typically found incidentally.

The most common symptoms of solid pseudopapillary tumors are abdominal pains, nausea, emesis, and sometimes palpable abdominal mass and jaundice – if the head of the pancreas is involved. In cases with compression of the stomach or the colon symptoms of obstruction may be observed. An asymptomatic course of the disease is not uncommon and diagnosis can be established incidentally, for example, following blunt abdominal trauma.

Complete resection is usually curative and so radical surgery is the treatment of choice. No adjuvant therapy is recommended.

## CASE PRESENTATION

**FIGURE 1:**
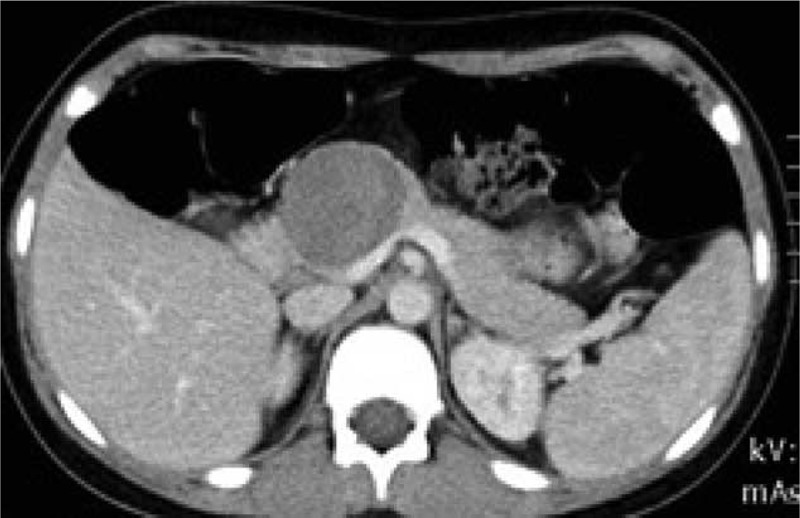
Computed tomography (CT) scan: the round shape mass located in the head of the pancreas.

**FIGURE 2:**
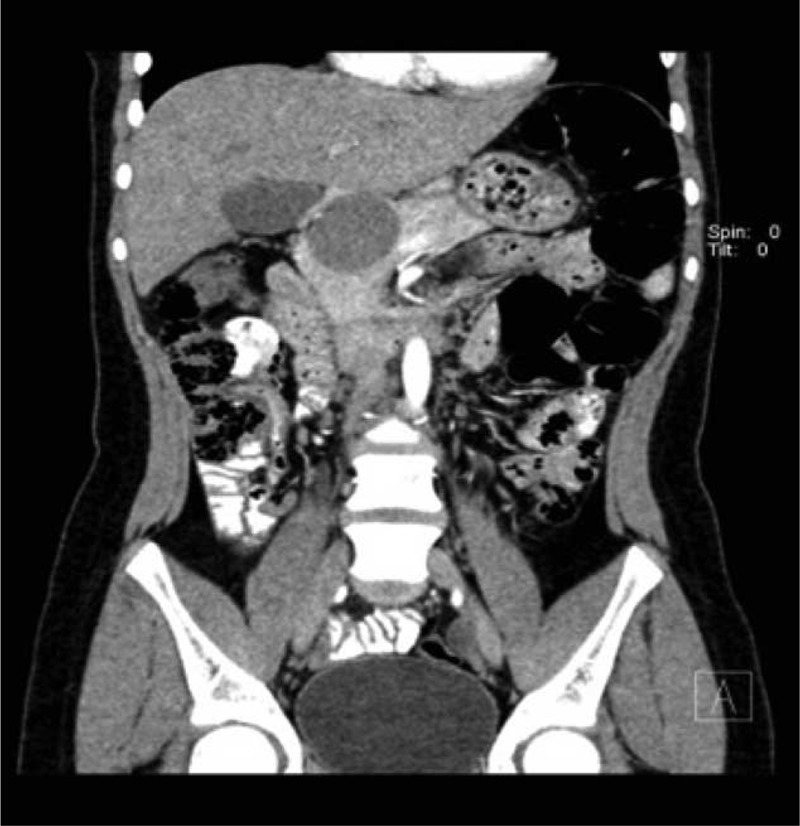
Computed tomography (CT) scan: Anterio-posterior AP reconstruction – tumor located in the head and body of the pancreas – visible margin of pancreatic head.

**FIGURE 3:**
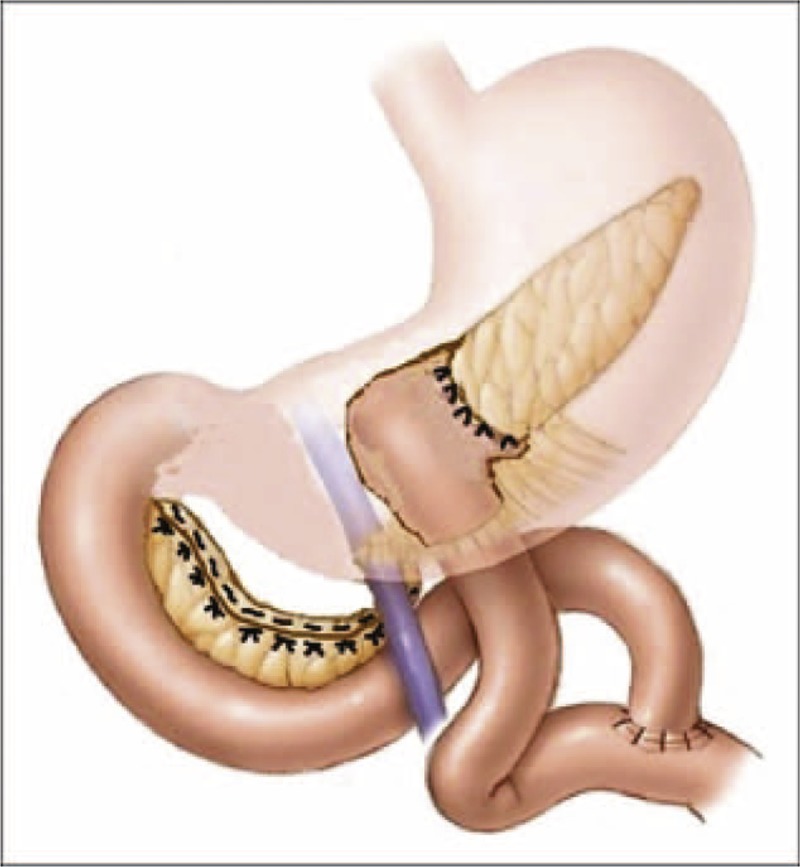
Frantz tumor resection. Central pancreatectomy with Roux-en-Y and end-to-end pancreaticojejunostomy (cuff anastomosis).

A 13-year-old girl was admitted to hospital because of a urinary tract infection. Ultrasonography revealed a 5 cm round mass located in the head and body of the pancreas. Computed tomography (CT) confirmed diagnosis and was characteristic of Frantz tumor (Figures 1 and 2). The patient was referred for surgical treatment. The location of the tumor indicated that pancreaticoduodenectomy should be the procedure of choice but favorable anatomical conditions during the operation allowed central resection of the tumor. The head of the pancreas from the duodenal border to the tumor border accounted for more than 3 cm, and allowed for 1 cm safe margins in resection from both sides of the tumor. The proximal pancreatic stump was over 7 with interrupted nonabsorbable 2–0 monofilament suture (Prolene 2-0 Ethicon). The distal part of the body and tail of the pancreas were anastomosed with a Roux-en-Y loop (double layer cuff anastomosis, Figure 3). Two closed suction drains were placed in the operative field. The early postoperative course was uncomplicated but on the 10th day after the operation active bleeding in the abdominal cavity was observed. Exploratory relaparotomy was performed and revealed active bleeding from a small arterial branch of the pancreatic artery in the connection between the Roux-en-Y loop and the tail of the pancreas. The bleeding was stopped with one monofilament stitch. In the postoperative course, pancreatic leakage was observed through the drains with amylase levels of more than 100,000 units and a loss of about 100 mL per day. The patient was managed with nothing by mouth and the leakage diminished during the course of a week, and stopped spontaneously 20 days after the 2nd operation. The serum level of amylase was below 500 units throughout the postoperative course. Pathological investigation confirmed epithelial solid papillary cystic neoplasm, and no adjuvant therapy was recommended. Control CT investigation did not reveal any relevant lesion, and the patient was discharged from hospital 30 days after the operation. The time of observation is 3 years. Informed consent was given by the patient for using her clinical data.

## DISCUSSION

The surgical treatment of pancreatic tumors includes various types of pancreatic resection, such as pancreaticoduodenectomy (Whipple operation), left pancreatectomy (with removal of the spleen in difficult cases), and total pancreatectomy. The type of procedure depends on the location of the tumor. Extensive resection procedures are justifiable in the surgical treatment of malignant tumors of the pancreas. These extensive procedures are connected with the removal of a large part of an organ, and they are associated significantly with the impairment of the exocrine and endocrine organ function.^3–7^ In benign, low-grade tumors or borderline tumors some types of excision procedures (leading to substantial impairment of the organ function) seem to be too extensive, given the consequences of resection. Middle pancreatectomy (central pancreatectomy [CP]) was performed for the first time with an oncologic indication in 1984 by Dagradi and Serio,^3,6^ and in recent years there has been growing interest in parenchyma-sparing pancreatic surgery in borderline, benign or low-grade malignant tumors. CP, duodenum-preserving pancreatic head resection (DP-PHR), and pancreatic head resection with or without segmental duodenectomy are well described in adults but only a few cases are reported in children. Careful patient selection is crucial for the use of these new techniques.^3,4,8–11^

In the presented patient the CT scans and ultrasound indicated that the lesion was located in the head of the pancreas, and the patient and the parents were informed that the range of the resection would be dependent on the anatomical condition assessed during the operation. The gastro-colic ligament was divided and the 5 cm round mass of the tumor was localized. The pancreatic tissue from both sides of the mass was tunneled. The proximal border of the pancreas was cut with a ligasure device and additionally oversewn with single monofilament nonabsorbable suture 2–0. The distal part of the pancreas was cut with a scalpel. The oncologic margin from both sides was acceptable for the lesion. Then the tumor was separated along a posterior plane from the superior mesenteric/portal vein confluence and removed. The reoperation resulted in the creation of a pancreatic fistula in the place of anastomosis with a Roux-en-Y loop. In the literature, the main problem after CP is the creation of a pancreatic fistula arising from the pancreaticoenterostomy site or from the closed pancreatic proximal stump. The risk of a pancreatic fistula is slightly higher than in the classical Whipple procedure, but the benefit of the preservation of the duodenal loop and maximal preservation of the pancreatic parenchyma is crucial, especially for children with long survival expectancy.^12–14^

An alternative method of anastomosis is the connection of the distal pancreatic stump with the posterior gastric wall. The procedure is technically easier because it reduces the number of anastomoses, but in our opinion the outflow of basic pancreatic juice into an acid gastric environment needs further investigation, especially in children. The authors chose anastomosis with Roux-en-Y strictly on the basis of personal experience.

In conclusion, progress in pancreatic surgery, especially in children, allows less radical options for the reason that preservation of endocrine and exocrine function is very important and protects them, especially from insulin-dependent diabetes in the future.
